# CSF-Biomarkers in Olympic Boxing: Diagnosis and Effects of Repetitive Head Trauma

**DOI:** 10.1371/journal.pone.0033606

**Published:** 2012-04-04

**Authors:** Sanna Neselius, Helena Brisby, Annette Theodorsson, Kaj Blennow, Henrik Zetterberg, Jan Marcusson

**Affiliations:** 1 Department. of Orthopaedics, Sahlgrenska University Hospital, Gothenburg, Sweden; 2 Institution For Clinical Sciences, The Sahlgrenska Academy at University of Gothenburg, Sweden; 3 Neurosurgery Section, University Hospital in Linköping, Linköping, Sweden; 4 Institution of Clinical and Experimental Medicine, Linköping University, Linköping, Sweden; 5 Clinical Neurochemistry Laboratory, Department of Psychiatry and Neurochemistry, Sahlgrenska University Hospital, Gothenburg, Sweden; 6 Institute of Neuroscience and Physiology, The Sahlgrenska Academy at University of Gothenburg, Gothenburg, Sweden; 7 Geriatric Section, University Hospital in Linköping, Linköping, Sweden; Hangzhou Normal University, China

## Abstract

**Background:**

Sports-related head trauma is common but still there is no established laboratory test used in the diagnostics of minimal or mild traumatic brain injuries. Further the effects of recurrent head trauma on brain injury markers are unknown. The purpose of this study was to investigate the relationship between Olympic (amateur) boxing and cerebrospinal fluid (CSF) brain injury biomarkers.

**Methods:**

The study was designed as a prospective cohort study. Thirty Olympic boxers with a minimum of 45 bouts and 25 non-boxing matched controls were included in the study. CSF samples were collected by lumbar puncture 1–6 days after a bout and after a rest period for at least 14 days. The controls were tested once. Biomarkers for acute and chronic brain injury were analysed.

**Results:**

NFL (mean ± SD, 532±553 vs 135±51 ng/L p = 0.001), GFAP (496±238 vs 247±147 ng/L p<0.001), T-tau (58±26 vs 49±21 ng/L p<0.025) and S-100B (0.76±0.29 vs 0.60±0.23 ng/L p = 0.03) concentrations were significantly increased after boxing compared to controls. NFL (402±434 ng/L p = 0.004) and GFAP (369±113 ng/L p = 0.001) concentrations remained elevated after the rest period.

**Conclusion:**

Increased CSF levels of T-tau, NFL, GFAP, and S-100B in >80% of the boxers demonstrate that both the acute and the cumulative effect of head trauma in Olympic boxing may induce CSF biomarker changes that suggest minor central nervous injuries. The lack of normalization of NFL and GFAP after the rest period in a subgroup of boxers may indicate ongoing degeneration. The recurrent head trauma in boxing may be associated with increased risk of chronic traumatic brain injury.

## Introduction

Violence against the head is a common event in a number of different sports and may result in sports-related Traumatic Brain Injury (TBI). In about 26% of all patients with head injuries seeking medical help at the emergency room the trauma was reported to be sports-related [1]. Terms used for mild, acute head injuries are concussion or mild traumatic brain injury (MTBI). The diagnosis of MTBI today is established by symptoms and clinical evaluation including a detailed neurological examination with balance testing and cognitive function [2], [3]. The management of mild head injuries is admission for observation and/or discharging after a normal computer tomography (CT) scan [4]. No information about the size or grade of the MTBI can be given and the time point when to return to sport is commonly decided using the Zurich 2008 Consensus Statement Return-to-Play protocol [5]. One problem with this protocol is that it is only useful when the patients have symptoms with a certain severity grade (e.g. clearly classified as a concussion).

Relatively little is further known about the late effects of multiple MTBIs. Epidemiological and animal studies have suggested association between repeated MTBIs/concussions and long-term consequences in form of chronic traumatic brain injury (CTBI) [6], [7], [8]. Several concussions have been demonstrated to lead to slower recovery [9] and young brains have been described to be more sensitive and needing a longer time for recovery after a MTBI compared to adults [10].

In association to the lack of objective diagnostic tools for MTBI the acute and long-term effects of olympic (amateur) boxing on the brain are debated. Several studies have failed to prove any signs of brain damage associated with olympic boxing [11], [12], [13] whereas the harmful long-term effects of professional boxing in form of dementia pugilistica have been known since 1928 [14], [15]. In boxing, acute TBI can be caused by structural brain injuries such as subdural haematoma, intracerebral haemorrhage or possibly by repeated MTBI/concussions [16]. MTBI may be caused by knock-out (KO) with loss of consciousness or by the cumulative effect of translational and rotational punches to the head [17]. These types of forces can result in cortical damage and diffuse axonal injury (DAI), which eventually may lead to CTBI [18], [19].

The relation between CTBI and Alzheimer's disease is debated. Neuropathologically, both conditions are characterized by neurofibrillary tangles and amyloid-containing plaques but the location and relative abundance of the changes differ; CTBI patients generally have more tangles than plaques preferentially involving the superficial cortical layers [20].

Presently, no objective diagnostic test, such as radiological or laboratory measurements to diagnose, grade and monitor TBI or early stages of CTBI, is in clinical use. CT (computed tomography), MRI (magnetic resonance imaging) and EEG (electroencephalography) are not sensitive enough and neuropsychological tests without a baseline measurement are difficult to interpret [11], [12], [13]. Analysis of CSF biomarkers can hopefully help us understand the pathology of a MTBI at the cellular level and may also have a role in clinical practice. To identify a reliable tool for grading of MTBI by individuals and further to use this tool in the evaluation of recovery, would be useful in return to sport guidelines.

Biomarkers for brain damage include neurofilament light protein (NFL), a marker of subcortical myelinated axons [21], total tau (T-tau), a marker of cortical axons [22], [23], tau phosphorylated at threonine 181 (P-tau_181_), a marker of tangle pathology [24], heart-type fatty acid binding protein (H-FABP), a marker of grey matter neurons [25], glial fibrillary acidic protein (GFAP) [26] and S-100B as markers of astroglial cells [27], [28] and the 42 amino acid isoform of amyloid β (Aβ1–42), marker of plaque pathology [23].

In the present study the relation between CSF biomarkers and boxing exposure in elite olympic boxers were investigated, both in the acute phase (within 6 days after a bout) and after a resting period (minimum 14 days), with the aim to search for tests to diagnose and monitor the effects of repetitive head trauma as in boxing.

## Methods

### Study population

The study was designed as a prospective prognostic follow-up study. Thirty olympic boxers competing at high national and/or international level were compared to 25 healthy, age-matched controls. All boxers had completed at least 45 bouts. This number was based on the regulation of the National Boxing Federation demanding an examination with MRI, CT or EEG every 50 bouts. The controls consisted of friends or relatives to the boxers, aiming to get controls with similar social background and education level. Exclusion criteria were athletes at elite level in sports where head trauma may occur, e.g., soccer, ice hockey and martial arts.

The regional ethical review board at Linkoping Health University, Sweden approved the study. Written informed consent was obtained from all participants.

### Questionnaire design and neurological examination

All participants filled in a questionnaire about medical history, medication, education, present occupation, information about previous concussions and quantification of alcohol and drug intake. Previous sports career was reported, to identify those who had trained in sports with risk of TBI. The questionnaire included a 10-question survey regarding previous and current symptoms of head and neck injuries based on a previous study [29]. The number of symptoms that had worsened over the last 5–10 years was added in a score. The boxers reported about their boxing career; fighting record, number of knock-out (KO) losses, number of Referee Stopping Contest losses due to several hard punches to Head (RSC-H), present weight class, duration of career, age at career start and age at first bout [15], [29]. Boxers gave an account for total amount of bouts the last week prior testing (1–3 bouts) and estimated these bouts as easy (1), intermediate (2) or tough (3). Three boxing experts independently (without any knowledge of the CSF biomarker concentrations) graded the boxers considering head trauma during total boxing career, 1 to 5 (1 is a boxer that has a low risk to receive blows to the head, according to boxing style, skills and the skills of the opponents. 5 is a boxer with high risk to receive repeated blows to the head). The total amount of bouts the last week before test A, the boxers own grading of the bouts and the mean of the expert grading were added in a score. This score was named “Boxing Exposure”. The aim was to calculate the total MTBI risk prior testing.

All participants underwent a neurological examination prior to lumbar puncture [9]. The neurological examination protocol included anamnestic questions about concussion symptoms, a general somatic status (general condition, examination of mouth and throat, heart, blood pressure, abdominal palpation, peripheral circulation and skin status) and a neurological status (orientation, alertness, speech function, cranial nerves 1–12, motor skills, balance, coordination, gate, sensibility testing and testing of reflexes). Magnetic resonance imaging (MRI) of the brain and neuropsychological testing (including among others short and long time memory, mental speed, recollection and cognitive testing) were performed in all participants without any structural injuries (haemorrhages, subdural haematomas) or other major findings observed. Detailed results of these investigations will be presented in a separate paper.

### CSF sample collection

The LP was performed at daytime, between 10 a.m. and 3 p.m, with the study objects in sitting position or laying on one side. For the first 18 objects a Quincke Type Point spinal needle (22 Gauge) were used, but since a few of the study objects suffered from postspinal headache, the needle was changed to a Sprotte (24 Gauge). Thereafter no more postspinal headache occurred. For each study object 5–10 ml CSF was collected in a polypropylene tube (Sarstedt, Nümbrecht, Germany), gently mixed to avoid gradient effects, aliquoted and stored at −80°C pending analysis. LPs were performed twice in the boxers: First LP 1–6 days after a bout (test A) and the second without exposure to bouts or training with blows to head for at least 14 days (test B). The control subjects underwent one LP.

### Biomarker analysis

NFL and GFAP were analysed using previously described ELISA methods [21], [30]. The detection limit of the NFL ELISA was 125 ng/L. CSF total tau (T-tau), tau phosphorylated at threonine 181 (P-tau181), and Aβ1–42 levels were determined using xMAP technology and the INNOBIA AlzBio3 kit (Innogenetics, Zwijndrecht, Belgium) as previously described [31]. S-100B was determined by an electrochemoluminescence immunoassay using the Modular system and the S100 reagent kit (Roche Diagnostics). H-FABP was measured using a commercially available ELISA method (Hycult Biotechnology, Uden, The Netherlands), following the instructions from the manufacturer.

Intra-assay coefficients of variation were <10% for all assays. All samples were analysed on one occasion to eliminate any inter-assay variability.

### Statistics

Differences between boxers and controls for the marker variables were tested by Student's t-test except for NFL where the non-parametric Mann Whitney U test was used (since many of the samples were below the detection limit <125 ng/L, a parametric test could not be used.) For the boxers, differences between time point A and B were compared using a paired sample T-test for all biomarkers except NFL where the non-parametric Wilcoxon signed rank test was used. For the boxers, differences between time point A and B were compared using a paired test. Regression analysis was used as an exploratory tool to explain variation of the marker values as a function of different factors. Bayesian Model Selection was used to identify the best predictive model [32]. Strength of association between the markers and “Boxing Exposure” was assessed by Spearman's rank correlation. Statistical analysis was carried out with SPSS 17.0 and R 2.10.

## Results

Two participants with complications after the lumbar puncture (back pain and headache, respectively) and two without complications declined follow-up.

### Questionnaire design and neurological examination

**Figure 1:**
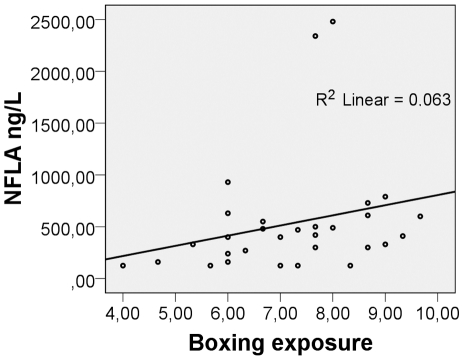
Cerebrospinal fluid concentrations of NFL in boxers test A correlate with Boxing Exposure. Boxing Exposure is a score that was constructed to calculate the total risk for traumatic brain injury before testing. It consists of three factors: 1. The total amount of bouts the last week before test A (1–3), 2. The boxers own grading of the bouts (easy (1), intermediate (2) or tough (3) and 3. The mean of the expert grading (3 boxing experts graded the boxers considering head trauma during total boxing career, 1 to 5). The results of these three factors were added in the boxing exposure score. Neurofilament light protein (NFL) analysed in cerebrospinal fluid (CSF) after bout (test A) correlated with Boxing Exposure, R = 0.396, p = 0.030.

The questionnaire about medical and social history and the 10-question survey were similar between boxers and controls (table 1). It is to be observed that none of the boxers suffered from loss of consciousness during their last bout before test A. Only one of the boxers reported concussion related symptoms after the bout (in this case headache) at the clinical examination, but the medical and neurological examination was normal in all subjects, GCS 15. There was no correlation between age or the risk factors listed in table 2 and brain injury markers when using a multiple regression model but “Boxing Exposure (BE)” gave a positive NFL (test A) correlation R = 0.396, p = 0.030 (fig. 1).

**Table 1:** Baseline details of boxers and controls.

	BOXERS	CONTROLS
Number	30	25
Age, years	22 (17–34)	22(17–30)
Sex:		
Male	28	20
Female	2	5
Education:		
Primary School	13%	20%
High School	67%	64%
University	20%	16%
Career:		
Unemployed	20%	16%
Student	33%	36%
Work	47%	48%
Other sports trained where trauma against head can occur (years).		
0	20%	28%
1–5	60%	28%
6–10	20%	20%
11–16	0%	24%
Concussions (yes)	17%	16%
Range	1–2	1
Neurological examination		
Normal	100%^1^	100%
10- question survey^2^		
Mean number ± SD	1.60±1.83	1.6±1.71
Alcohol intake		
NO	40%	16%
<once per week	50%	56%
Once per week	3%	20%
>once per week	7%	8%
Drugs (marijuana, hashish)	0%	12%

1 One of the boxers was born without the smell sense and had been evaluated according this.

2 Neuropsychological intervention with 10 different symptoms of head and neck injury based on a previous study [29]. Worsening of the number of symptoms the last 5–10 years was evaluated.

**Table 2:** Boxer's details and risk factors for brain injury.

**AGE** (years)			
Test A^1^	30 boxers, mean 22, range 17–34		
Test B^2^	26 boxers, mean 24, range 17–34		
**AGE, WHEN THE BOXING CAREER STARTED**	Mean 13.9 (median 14) Range 7–19		
**AGE AT FIRST BOUT** (years)	Mean 14.6 (15) 10–19		
**DURATION OF BOXING CAREER** (years)	Mean 7.2 (8) 3–13		
**DIPLOMA BOUTS** 3 (number)	Mean 17.5 (10) 0–57		
**REGULAR BOUTS** (number)			
Test A	Mean 74 (61) 47–168		
Test B	Mean 92 (79) 47->200		
**WINS** (%)			
Test A	Mean 70 (71) 25–92		
Test B	Mean 68 (70) 25–92		
**KO- LOSSES** (knock-out)			
One	8 (27%)		
Three	1(3%)		
**RSC-H** 4 **LOSSES**			
One	5 (17%)		
Two	1(3%)		
**WEIGHT CLASS** (kg)	Mean 70.2 (69) 54–91		
**BOXING STYLE**			
Defensive boxer	7%		
Counterattack boxer	66%		
Attack boxer	27%		
**EXPERT SCORING** 5			
Mean score ≤2.0	7%		
Mean score 2.1–3.9	74%		
Mean score ≥4.0	20%		
**DAYS SINCE LAST BOUT**			
Test A	Mean 2.7 (2) 1–6		
Test B	Mean 148 (26) 14–760		
**BOXING EXPOSURE (BE)**			
Scoring of last bout^6^	20%, Easy	47%, Intermediate	33%, Tough**
Number of bouts^7^	40%, 1 bout	40%, 2 bouts	20%, 3 bouts**
Sequelae (headache)^8^			3% (1 boxer)**

1 1–6 days after a bout;

2 A rest period of a minimum of 14 days;

3 Boxing at age 10–14 years without hard punches;

4 Referee Stops Contest due to hard blows against head;

5 Three experts graded the boxers 1 to 5, independently, (from low to high head trauma exposure considering total boxing career);

6 The boxers scored their last fight as easy, intermediate or tough;

7 Number of bouts in a row (maximum one per day) for the test A;

8 If a boxer experienced some sequelae after the last bout;

** Boxers with increased risk for MTBI.

### Biomarkers for neuronal injury

**Figure 2:**
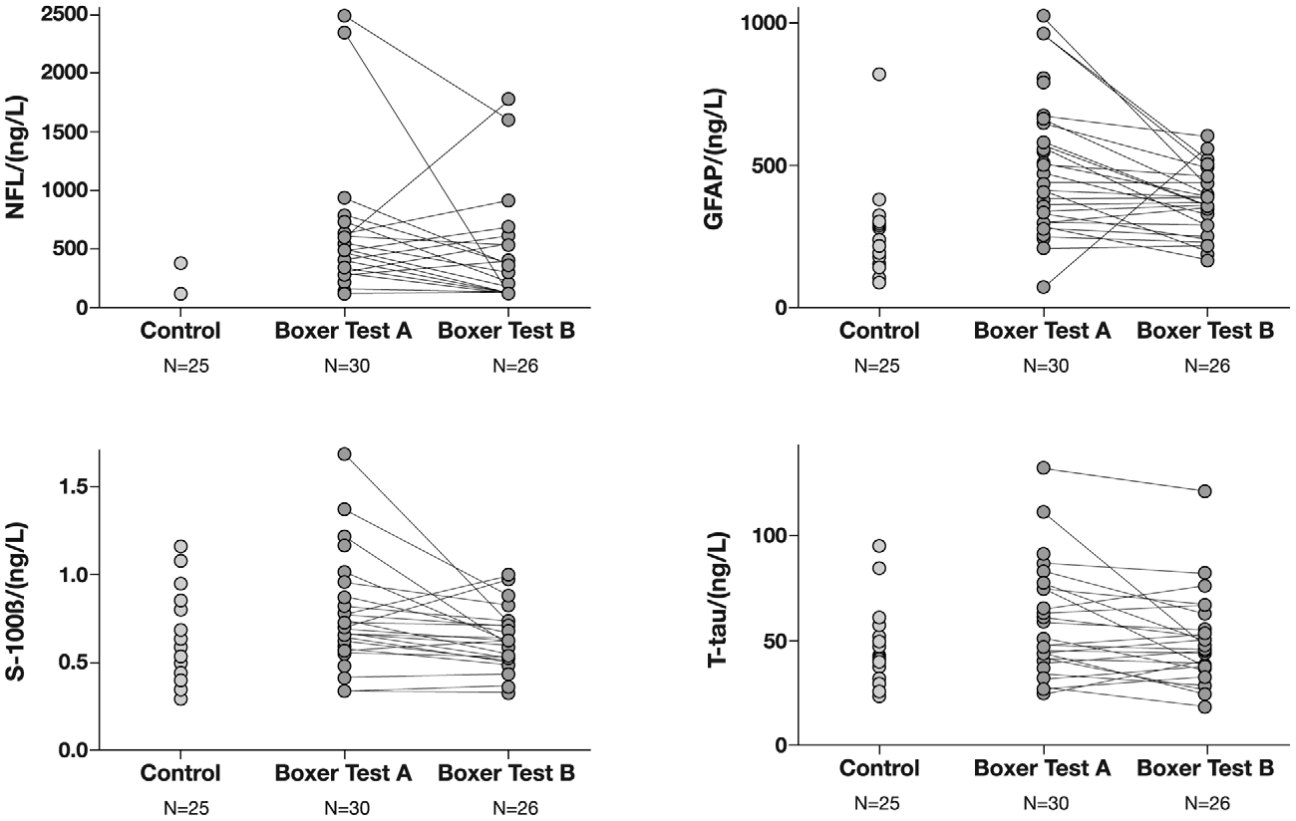
The individual change of CSF biomarker concentrations for boxers vs controls. Cerebrospinal fluid (CSF) was collected from controls once. In boxers the CSF was collected 1–6 days after a bout (A) and after a rest period of at least 14 days (B). The figure illustrates the individual change of neurofilament light protein (NFL), glial fibrillary acidic protein (GFAP), S-100B and total-tau (T-tau). NFL in all controls expect one (380 ng/L) were below the detection limit of 125 ng/L, expressed as 125 ng/L on the chart. All controls had GFAP levels between 90–380 ng/L except the subject with elevated concentration of NFL.

**Figure 3:**
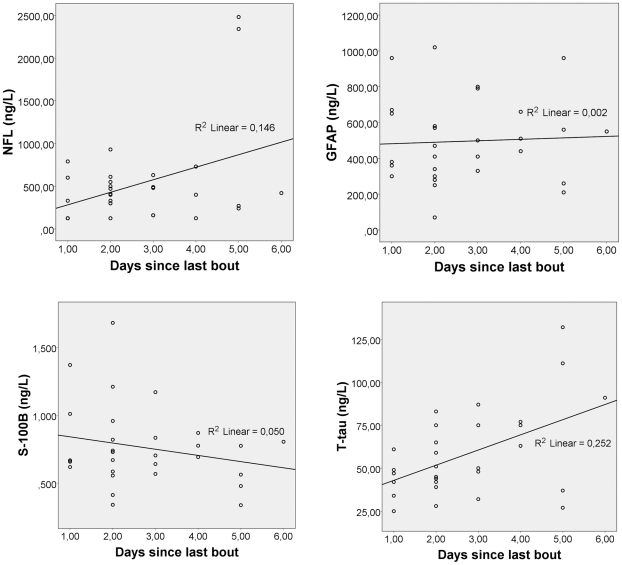
CSF biomarker concentrations vs days in test A. Cerebrospinal fluid concentrations of NFL, GFAP, T-tau and S-100B from the boxer group after bout (test A) plotted vs time in days after their last bout. For NFL and T-tau increasing concentrations are seen with time. The opposite is observed for S-100B and no relationship was seen between GFAP and time when the samples were collected.

The boxers had elevated concentrations of NFL at test A and B compared to controls (Table 3, fig. 2). Only five of 30 boxers had NFL below the detection limit of 125 ng/L after a bout, which is considered normal for this age group [21] and the rest had >125 ng/L. One of the controls had a concentration of 380 ng/L, all the others below 125 ng/L. At follow-up, 13 of 25 boxers had NFL>125 ng/L (fig. 2). Regression analysis for test A showed that NFL level increased by 147 ng/L per day between days 1–6 after a bout (±1 SE 67.0), t = 2.190, p = 0.037 (fig. 3). The two boxers who had the highest values at the first test, 2340 ng/L and 2480 ng/L, were tested 5 days after a bout. After a resting period of 14 days, the concentration was 125 and 1600 ng/L, respectively (fig. 2). Their Boxing Exposures (BE) were 1 and 2 tough bouts and expert score 4.0 and 5.0, respectively. The only boxer reporting sequelae (headache) had “BE”: 3 bouts, tough and mean expert score of 3.7. Test A performed a day after the last bout revealed a NFL concentration of 600 ng/L, which increased to 1780 ng/L 15 days later (fig. 2). In total, 7 of the boxers with pathological concentrations of NFL at test A had even higher values at follow-up (fig. 2). Interestingly one of these had not been boxing for 360 days.

**Table 3:** Biomarker concentrations in CSF.

CSFBiomarker	Boxer Test A^1^ N = 30 Mean(range)SD ng/L	Boxer Test B^2^ N = 26Mean(range)SD ng/L	Controls N = 25Mean(range)SD ng/L	P-value		
				A vs. C	A vs. B	B vs. C
**NFL**	532(125–2480)553	402(125–1780)220	135(125–380)51	<0.001	0.072	<0.001
**GFAP**	496(70–1020)238	367(170–600)113	244(90–820)145	<0.001	0.011	0.001
**FABP**	407(108–1089)208	334(40–769)195	458(67–1383)271	0.45	0.07	0.07
**S-100β**	0.76(0.34–1.68)0.29	0.63(0.33–0.99)0.16	0.60(0.30–1.16)0.23	0.03	0.016	0.67
**Aβ1–42**	306(191–411)52	294(178–423)54	297(231–362)39	0.43	0.37	0.83
**T-Tau**	58(25–132)25	49(19–121)21	45(24–95)17	0.025	0.024	0.39
**P-Tau**	21(9–38)7	22(9–43)8	23(14–40)6	0.21	0.09	0.68

1 Test A: 1–6 days after last bout;

2 Test B: No boxing for at least 14 days. Statistical analyses were performed with parametric methods for all biomarkers except NFL where non-parametric method was used due to values below the detection limit.

Concentrations of T-tau were significantly higher at test A in boxers compared to controls (Table 3, fig. 2). The concentrations had normalized at follow-up, although one of the boxers with high concentrations of NFL (2480 ng/L) and GFAP (960 ng/L) after a bout also had high concentration of T-tau at the follow-up, 121 ng/L (fig. 2). Regression analysis showed that the concentration of T-tau increased by 8.8 ng/L per day (±1 SE 1.7), t = −2.477, p = 0.019 in the time span 1 to 6 days after a bout (fig. 3).

With regards to H-FABP, no significant differences were found between boxers and controls (Table 3).

### Biomarkers of astroglial injury

The boxers had elevated concentrations of GFAP at both test A and B compared to controls (Table 3, fig. 2). All controls had GFAP levels between 90–380 ng/L except the subject with elevated concentration of NFL (fig. 2). This individual had a GFAP concentration of 820 ng/L, which is considered abnormal for this age group [30]. Eighteen of 30 boxers had GFAP concentrations ≥410 ng/L (the value calculated by mean concentration of the controls, without the outlier, plus 2SD) at test A including three boxers that were lost to follow-up. Seven boxers still had GFAP concentrations ≥410 ng/L at follow up (fig. 2). One boxer had a GFAP concentration of 70 ng/L at the first test and 560 ng/L at follow up (fig. 2). In between the two tests, the boxer had fought 84 bouts. Test A by the boxer with the highest value, 1020 ng/L, was taken 2 days after a series of 3 bouts, scored easy and with a mean expert score of 2.0. The corresponding NFL concentration was 930 ng/L. The two boxers with the highest NFL concentrations at test A, had corresponding GFAP of 560 and 960 ng/L (test A) and 290 and 520 ng/L (test B), respectively.

Concentrations of S-100B were significantly increased after a bout, but normalized at follow up, compared to controls (Table 3, fig. 2).

### Markers of neurofibrillary tangle and plaque pathology

**Figure 4:**
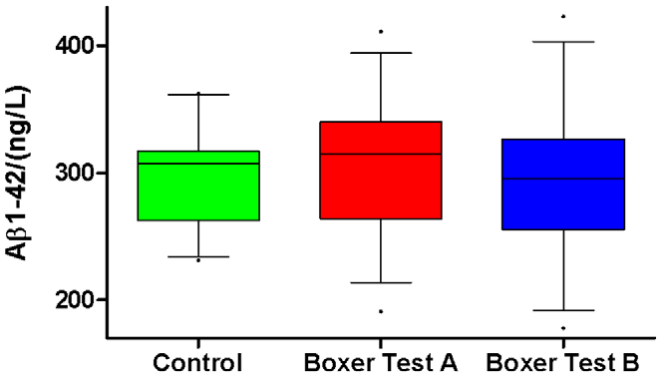
Aβ1–42 shows a larger variation in the boxers vs controls. Cerebrospinal fluid (CSF) was collected from the controls once. The boxers were tested 1–6 days after a bout (A) and after a rest period without exposure to bouts or training with blows to head for at least 14 days (test B).

No significant differences between boxers and controls were found for P-tau and Aβ1–42, although the variability for Aβ1–42 was larger in the boxer group, −38% to +100%, mean +16% ±SD 37 (fig. 4). The boxer with the lowest Aβ1–42 concentration at test A and B showed no pathological CSF concentrations of NFL or GFAP. This boxer had a low education and career level, had trained soccer for 8 years and started boxing 8 years ago at an age of 12 years. The boxing career included 40 diploma bouts and 51 normal bouts. Five of the boxers showed 16–43% decreases of Aβ1–42 at the follow up whereas 2 of the boxers had increased concentrations (15 and 76% respectively).

## Discussion

This study has gathered the largest sample so far of amateur elite boxers and matched controls to examine a possible relationship between MTBI in amateur boxing and CSF biomarkers.

Although only one of the boxers had self-reported symptoms of a possible concussion and the clinical examination was normal in all the boxers, the data demonstrate that concentrations of NFL, GFAP, S-100B and T-tau in CSF were increased within 6 days after a bout in more than 80% of the amateur boxers indicating acute axonal and neuronal damage. NFL, GFAP and T-tau are specific markers for damage of the central nervous system and increased concentrations of NFL [21], [33] and GFAP [26] have previously been found both after acute and chronic brain injuries caused by different types of trauma. Both NFL and GFAP further remained significantly elevated after a resting period of at least 14 days (test B) in the boxers compared to the controls in the present study. Most of the boxers with increased NFL and GFAP concentrations at test B had fought many bouts, both before test A, and between the two tests, which may have resulted in a cumulative effect. One previous study has analysed CSF biomarkers in relation to boxing, where 14 boxers compared to 10 controls showed elevated concentrations of NFL, GFAP and T-tau 7–10 days after a bout. Only NFL remained elevated at a 3 month follow-up and correlation was found between CSF concentration of NFL and an injury severity score [34]. Also in the present study, a correlation was found between a composed boxing exposure index and NFL, but construction of such an index is difficult since the development of a possible brain injury could potentially be related to all the risk factors listed in table 2. Concentrations of NFL and T-tau gradually increased with time during the first 6 days after trauma. These results are in accordance with previous findings in patients with TBI [22]. No concentration peak for GFAP was found, but the boxer with the reported concussion had among the highest concentration levels of GFAP one day after the bout and the levels had decreased from 960 to 500 ng/L at follow up, 15 days after the bout. More studies are needed to investigate if NFL and GFAP correlate with the size of injury.

Our study also revealed higher concentrations of S-100B after a bout compared to controls.

S-100B is a calcium binding protein physiologically produced and released by astrocytes and other glial cells in the central nervous system (CNS) [35]. Outside the nervous system it can be found in adipose tissue, muscle and skin [36]. S-100B increases after brain injury and remains elevated for up to 5 days in CSF, with a peak at day one [23]. The concentrations have been observed to correlate with brain injury severity [37]. In serum, S-100B increases after MTBI [27] and S-100B levels have also been found to rise after physical activity such as marathon running. S-100B released from skeletal muscle has a relatively short half-life in serum and the levels are back to normal levels within 20 hours [38]. To our knowledge no studies have shown transport of S-100B from serum to CSF, why analysis of S-100B in CSF most likely reflects the true cerebral S-100B concentration [37]. The role of released S-100B after TBI is not clearly understood but it might have both neurotrophic and neuroprotective functions, or simply reflect injury-related release [39].

Little is known about the dynamics of Aβ1–42 in CSF but recently the concentrations of Aβ1–42 were demonstrated to correlate with neurological status after acute brain injury. The concentrations were consistent between different sampling occasions in healthy patients but decreased after a brain injury and increased when the neurological status improved [40]. Therefore, even though no statistic differences were found, one explanation for the relatively large variation in Aβ1–42 concentrations between test A and B in some of the boxers may be a traumatic brain injury at some stage. In a similar manner the large variation of P-tau with indications of gradual increase in the boxers may be an early sign of neurofibrillary tangle build-up.

The strength of this study is the very well matched baseline parameters for boxers versus controls. The only difference was a longer career of other sports where trauma against head occurs in controls compared to boxers (24% vs 0%) (table 1). Interestingly, the only control subject with elevated levels of NFL and GFAP was among those who had a previous sport career including head trauma. A previous study on CSF biomarkers in soccer players did not find any evidence of TBI in that group [41]. A limitation of this study is the variation of time points for CSF sampling. The ideal had been to have test A and B collected at the same time points for all participants. It would also have been preferable with a longer rest period before the B sampling. This was not possible since we had to adapt the samplings to the boxers' schedules in order to perform the study.

In conclusion, this study shows that the repetitive head trauma occurring in olympic boxing may induce changes in CSF NFL, GFAP, T-tau and S-100B, even without anamnestic or clinical symptoms of a concussion or traumatic brain injury. These changes suggest minor central nervous system injuries. It seems that most of the acute injuries can recover with rest but without an appropriate rest period there might be a risk for cumulative injury. The length of the rest period needed seems either to be individual or is correlated to the size of the injury.

Further studies are needed to evaluate if nervous system injury biomarkers in CSF may be useful as an evaluation tool in clinical praxis in the diagnosis and grading of a concussion/MTBI and as part of return to sport guidelines. Future studies of interest include closer monitoring of boxers at different early time-points after repeated head trauma at bouts, long-time follow-up of boxers and also monitoring of CSF biomarkers in patients attending emergency departments due to a concussion where the clinical examination is normal.
